# Understanding the off-loading mechanism of adenosylcobalamin by *Cupriviadus metallidurans* adenosyltransferase from *C. metallidurans* Isobutyryl-CoA Mutase Fused

**DOI:** 10.1063/4.0000928

**Published:** 2025-10-27

**Authors:** Jayoh Amurao Hernandez

**Affiliations:** MIT, Cambridge, MA, USA

## Abstract

Enzymes are Nature's highly efficient catalysts, driving the metabolism of diverse substrates essential for sustaining life across all biological kingdoms. Enzymes often require cofactors that range from simple metal ions to complex organometallic cofactors. One such important metallocofactor is adenosylcobalamin (AdoCbl), which catalyzes radical-based chemical reactions. AdoCbl is crucial for the maturation of mutases, a family of enzymes that mediates the rearrangement of the carbon skeletons of various amino acids, cholesterol, and fatty acids. Methylmalonyl-CoA mutase (MCM) is a human mutase that depends on two metallochaperones: methylmalonic aciduria type A (MMAA), a G- type protein, and adenosyltransferase (ATR). Mutations or deletions in any genes related to MCM that affect cofactor loading and transfer can lead to methylmalonic aciduria, a potentially fatal inborn error of metabolism. Due to the rapid turnover of MCM, the AdoCbl cofactor is prone to damage. When this occurs, ATR extracts the damaged cofactor from MCM, repairs it, and then reattaches the regenerated AdoCbl to the mutase. The mechanism of off-loading, transfer, and reloading of the cofactor has remained molecularly and structurally elusive, as the MCM-MMAA-ATR complex is a transient three-protein assembly that is difficult to capture. However, isobutyryl-CoA mutase fused (IcmF) from *Cupriviadus metallidurans*, a homologue of MCM, presents an opportunity to study this process. In IcmF, the G-protein MMAA is fused directly to the mutase, forming a simpler two-protein complex (IcmF-ATR) that circumvents the challenges posed by the MCM system. Our goal is to understand how ATR recognizes the damaged cofactor, takes it from IcmF, and subsequently delivers the repaired cofactor to the enzyme. Leveraging crystal structures of *Cm*IcmF and *Cm*ATR determined in this work, we are examining two histidine residues (H872 and H993), which are hypothesized to serve as intermediate binding sites in the cofactor transfer process. To monitor the activity, we are using an assay based on UV-vis spectroscopy to measure the kinetics of the cofactor transfer process. Ultimately, this research aims to deepen our understanding of AdoCbl-dependent enzyme maturation and provide insight into the molecular basis of methylmalonic aciduria.

